# Functional‐Structural Correlates in Achalasia: The Relationship of Esophageal Pressurization and Anatomy

**DOI:** 10.1111/nmo.70180

**Published:** 2025-10-09

**Authors:** John E. Pandolfino, Eric Goudie, Jacob M. Schauer, Domenico A. Farina, Leya Chambo, William Ravich, Linda Kelahan, Dustin A. Carlson

**Affiliations:** ^1^ Kenneth C. Griffin Esophageal Center of Northwestern Medicine, Department of Medicine, Division of Gastroenterology and Hepatology, Feinberg School of Medicine Northwestern University Chicago Illinois USA; ^2^ Division of Thoracic Surgery, Department of Surgery Université de Montréal Montreal Quebec Canada; ^3^ Division of Biostatistics, Department of Preventive Medicine, Feinberg School of Medicine Northwestern University Chicago Illinois USA; ^4^ Section of Digestive Diseases, Department of Internal Medicine Yale University School of Medicine New Haven Connecticut USA; ^5^ Department of Radiology, Feinberg School of Medicine Northwestern University Chicago Illinois USA

**Keywords:** dysphagia, mechanics, motility, remodeling

## Abstract

**Background and Aims:**

Achalasia subtypes are classified by high‐resolution manometry (HRM) based on esophageal pressurization and contractility patterns, while esophagram‐based classifications emphasize esophageal anatomy. We aimed to evaluate the relationship between esophageal pressurization on HRM and esophageal anatomy on esophagram among patients with untreated achalasia.

**Methods:**

Adult patients with treatment‐naïve achalasia that completed HRM and esophagram were included. HRM achalasia subtypes were determined by the Chicago Classification with pan‐esophageal pressurization (PEP) measured among type I and type II achalasia. Anatomy on esophagram was assessed using the Brazilian (esophageal width) and Japanese Esophageal Society (JES; angulation/tortuosity) classifications.

**Results:**

222 patients, mean (SD) age 56 (16), 49% female were included. On HRM, 32% were type I, 53% were type II, and 15% were type III achalasia. Esophageal width and JES classification differed by HRM subtype (*p*‐values < 0.001) with type I (HRM) having greatest esophageal width (median (IQR) 5.1(4.0–6.0) cm) and most JES‐C 93% (14/15), while type III achalasia had the least (width 2.6 (2.0–3.0) cm) and 0 were JES‐C. Among type I and II achalasia, higher esophageal width was significantly correlated with lower median PEP and fewer swallows exceeding PEP thresholds of 10, 15, 20, or 30 mmHg.

**Conclusions:**

HRM subtypes and PEP on HRM correlated with esophageal morphology defined on esophagram. However, imperfect concordance between HRM and esophagram classifications suggests complementary value to assess achalasia disease stages related to disease chronicity and esophageal wall mechanics. Future investigations to facilitate combined assessment with HRM and esophagram may enhance achalasia phenotyping and treatment planning.

## Graphical abstract

High‐resolution manometry (HRM) Chicago Classification subtypes and panesophageal pressurization generally correlated (inversely) with esophageal morphology defined by width (Brazilian classification) and anatomic deformity (Japanese Esophageal Society classification) on esophagram in achalasia. However, overlap and discordance were also observed, suggesting value in the complementary application of HRM and esophagram in achalasia.

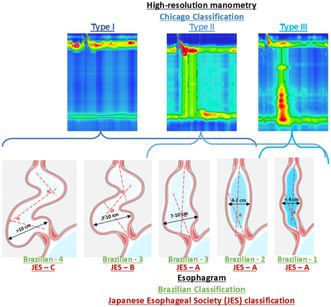

**Summary**

- Achalasia is a esophageal disorder in which esophageal nerve and muscle function is impaired which causes difficulty swallowing. This study compared classifications derived from two key tests used to evaluate achalasia esophageal anatomy on X‐ray (esophagram) with pressure patterns measured during high‐resolution manometry (HRM).
- Greater esophageal widening and twisting were associated with lower pressurization on HRM, suggesting that structural remodeling of the esophagus corresponds to more advanced stages of achalasia.
- Although related, anatomic and functional classifications did not always align, supporting the use of both imaging and manometry to provide a more complete assessment of disease severity and to inform treatment planning.

## 1 Introduction

Achalasia is subtyped on high‐resolution manometry (HRM) based on the presence (or absence) of pan‐esophageal pressurization (PEP) or premature contractility when there is also an elevated integrated relaxation pressure (IRP) and absent peristalsis. Type I (classic) achalasia and type II achalasia are both characterized by absent contractility and are then differentiated by the “absence” (type I) or presence (type II) of panesophageal pressurization (PEP). Type III (spastic) achalasia is characterized by the presence of premature contractility. These achalasia subtypes have been consistently reflected in updates of the Chicago Classification and carry interest related to prognostication of treatment response and directing treatment decisions (e.g., preferential use of surgical myotomy over pneumatic dilation for type III achalasia) [1, 2, 3, 4].

There has been ongoing interest and recognition in the relationship between esophageal anatomy, such as dilatation or tortuosity, and pressurization patterns on HRM since the initial HRM subtype descriptions [1, 5, 6]. As esophageal deformity and remodeling are associated with the chronic consequences of the achalasia disease process, ongoing appreciation of the impact of esophageal anatomy on achalasia disease severity is apparent [7, 8, 9]. Hence, achalasia has also been classified based on esophageal anatomy using barium radiography (esophagram) with the classification scheme described by the Japanese Esophageal Society (JES), Brazilian and Italian groups [5, 8, 9].

We hypothesized that the evaluation of the relationships between the HRM achalasia subtypes and the degree of PEP on HRM with esophageal anatomy may shed insights on esophageal wall mechanics and may have important implications for defining achalasia disease stage and potentially refining achalasia subtypes. Hence, we aimed to describe the relationship of esophageal pressurization (PEP) on HRM with esophageal anatomy defined on esophagram utilizing esophagram classifications among patients with treatment‐naïve achalasia.

## 2 Methods

### 2.1 Subjects

This retrospective cohort study included consecutive patients with idiopathic achalasia (HRM Chicago Classification subtypes I, II, or III) diagnosed from 2014 to 2023 who completed evaluation with baseline (pre‐treatment) HRM, pre‐treatment esophagram, and also had a follow‐up esophagram after treatment available. Baseline esophagrams completed without a formal timed‐barium esophagram (TBE) protocol were still included; however, non‐TBE esophagrams did not have column height measures for related analysis. This study focused on the baseline (pre‐treatment) esophagram and HRM, though post‐treatment esophagrams' availability was an inclusion criterion to facilitate future studies focused on treatment outcomes. Patients were retrospectively identified for inclusion from the Esophageal Center of Northwestern Medicine esophageal motility registry, which is prospectively maintained and includes adult patients (ages 18–89 years) who present to the Esophageal Center of Northwestern Medicine for evaluation and management of esophageal conditions including achalasia. Patients with a history of foregut surgery or pneumatic dilation prior to baseline HRM were excluded; Figure S1.

The study protocol was approved by the Northwestern University Institutional Review Board as minimal risk with a waiver of informed consent for retrospective analysis of deidentified patient data.

### 2.2 HRM Protocol and Analysis

**FIGURE 1:**
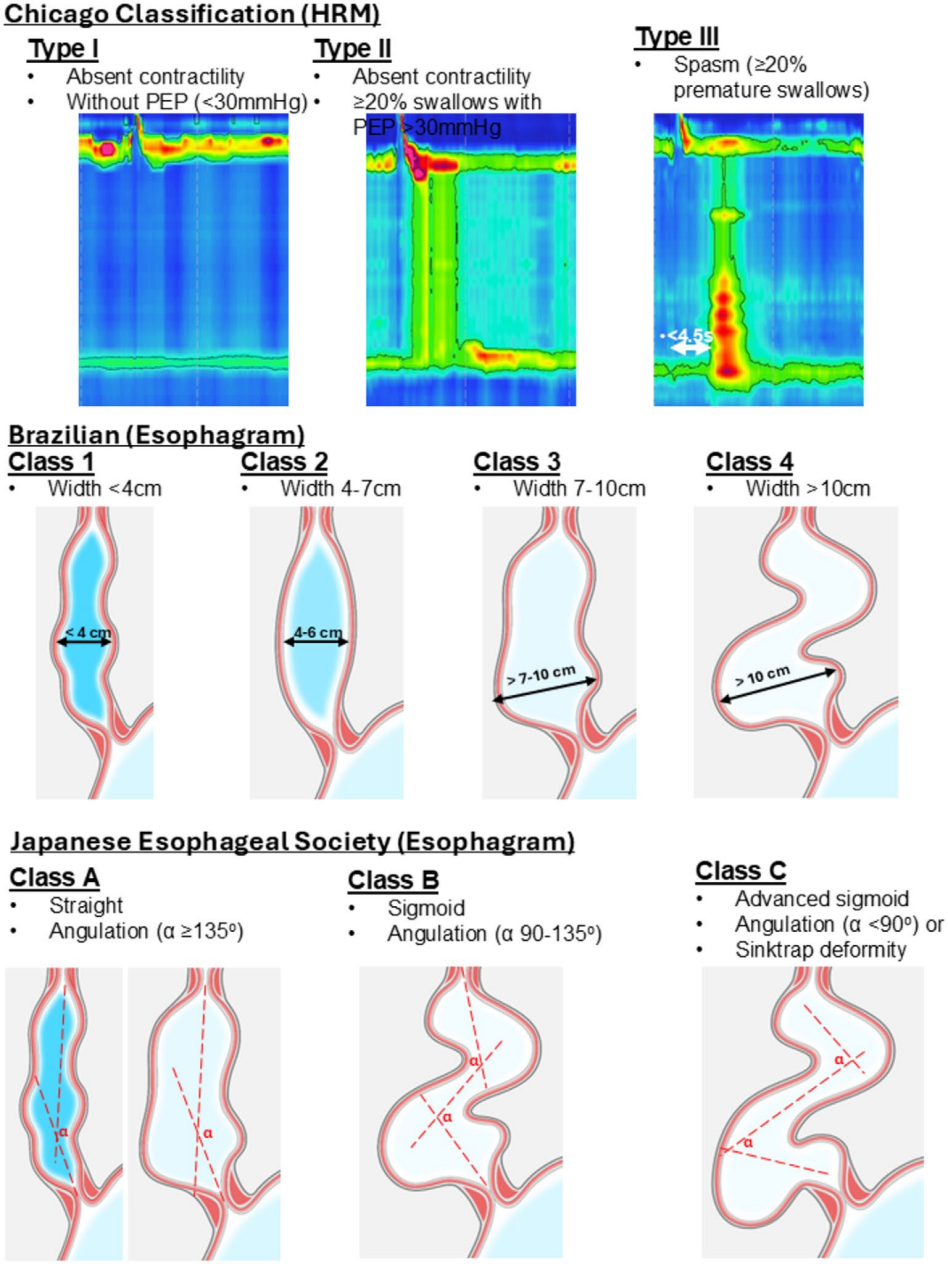
Criteria for achalasia classification schemes. High‐resolution manometry (HRM) criteria were applied to supine test swallows and also included an elevated integrated relaxation pressure (IRP) and absent peristalsis. Figure used with permission of the Esophageal Center of Northwestern.

HRM studies were completed after a 6‐h fast using a 4.2‐mm outer diameter solid‐state assembly with 36 circumferential pressure sensors at 1‐cm intervals (Medtronic, Shoreview, MN). The HRM assembly was placed transnasally and positioned to record from the hypopharynx to the stomach with approximately three intragastric sensors. After a 2‐min baseline recording, the HRM protocol was performed, including ten 5‐mL liquid swallows in a supine position and then five 5‐mL liquid swallows in an upright position [4]. Studies were analyzed using commercially available software (ManoView, Medtronic) according to the Chicago Classification; Figure 1 [2, 3, 4]. HRMs were reviewed for this study, blinded to clinical characteristics (including esophagram findings, treatment, and follow‐up/outcome details) to measure PEP values [4, 10]. For each supine test swallow among patients with type I and type II achalasia, the PEP value was measured as the pressure at which the first break of the strongest PEP occurred while scaling up the isobaric contour tool. PEP values were examined based on the number of swallows reaching various PEP thresholds (e.g., similar to how type II achalasia is defined by ≥ 2 swallows exceeding a PEP threshold of 30 mmHg) as well as the median PEP values from the 10 supine test swallows.

### 2.3 Barium Esophagram Protocol and Analysis

Barium esophagrams were performed in the upright position. For TBEs, patients consumed 200 mL of low‐density barium sulfate with anteroposterior images obtained at 1, 2, and 5 min [11]. Esophagrams were reviewed by study team members (EG, DF, LK), who were blinded to clinical characteristics, including HRM findings and treatment details. Esophagram analyses were performed using Visage software (San Diego, CA, USA) available through the Northwestern electronic medical record. For TBEs, the height of the barium column was measured vertically from the EGJ to the top of the barium column on the 1‐, 2‐, and 5‐min images.

For all esophagrams (TBE protocol or not), the maximum esophageal body width was measured at the greatest transverse dimension on review of all available esophagram images; these were applied to define the Brazilian classifications, ranging from Brazilian class 1 with the least esophageal dilatation to class 4 with the greatest dilatation; Figure 1 [9]. The previously described Italian classification was similar to Brazilian, with the exception of differentiating class 2 from 3 using a threshold of 6 cm and incorporating a sigmoid morphology to differentiate class 3 from 4 [5, 9]. We opted to utilize the Brazilian classification for this study to provide a classification focused on esophageal dilatation.

Qualitative impression of the esophageal body morphology was assessed as straight or sigmoid, and trajectory lines drawn along the esophageal long axis were utilized to assess the degree of angulation (i.e., esophageal tortuosity) to define the JES classifications (Figure 1). Trajectory lines were placed manually using the Visage software tools to measure the degree in angulation of the lines. JES class A indicated minimal esophageal distortion, JES class B intermediate, and JES class C indicated the most severe anatomy distortion [8]. Other morphological features such as corkscrew or sigmoid appearance were also recorded.

### 2.4 Statistical Analysis

Results were reported as mean (SD) or median (interquartile range) depending on the data distribution. Correlation of continuous variables was assessed using Pearson or Spearman's correlation, depending on data distribution. Groups were compared with the Chi‐square (*χ* ^2^) test for categorical variables and ANOVA/*t* tests or Kruskal‐Wallis/Mann–Whitney *U* for continuous variables, depending on the data distribution. Post hoc comparison testing was completed using a Bonferroni correction to address multiple comparisons. Unless otherwise specified, a 2‐tailed *p* value < 0.05 was considered to meet statistical significance.

## 3 Results

### 3.1 Subjects

222 patients, mean (SD) age 56 (16), 110 (49%) female, patients with achalasia were included; Table 1; Figure S1. There were 72 (32%) type I, 117 (53%) type II, and 33 (15%) type III achalasia by HRM/Chicago Classification subtypes. 74% of the baseline esophagrams were completed using a TBE protocol. Among the baseline TBEs, barium column height did not differ between HRM subtypes at the 1 min (*p* = 0.320) or 2 min (*p* = 0.404) measure, but did differ at 5 min (*p* = 0.028) with greater column heights in type II than in type III (adjusted *p*‐value = 0.022); Table 1. The 5‐min barium column height numerically appeared greater in type I than in type III achalasia, but this was not a statistically significant difference (adjusted *p*‐value = 0.132).

**TABLE 1:** Cohort characteristics and associations between HRM achalasia subtypes and esophagram classifications.

Characteristic	Total cohort	HRM‐Chicago Classification Achalasia subtypes		
		Type I achalasia	Type II achalasia	Type III achalasia
*N*/*n*	222	72	117	33
Age, mean (SD), years*	53 (16)	54 (15)	51 (17)	61 (15)
Sex, female, *n* (%)	110 (50)	39 (54)	57 (49)	14 (42)
On opioids, *n*/*n* ^a^ (%)*	8/149 (5)	1/49 (2)	3/82 (4)	4/18 (22)
*n* (%) completing TBE protocol	168 (76)	56 (77)	86 (72)	26 (77)
TBE column heights, median (IQR), cm^a^				
1 min	11.4 (8.0–17.3)	10.2 (5.6–15.8)	12.5 (8.9–16.3)	15.0 (0–21.6)
2 min	10.4 (6.4–16.9)	9.8 (5.5–15.0)	11.4 (7.0–17.0)	12.0 (0–19.3)
5 min*	9.3 (4.9–13.2)	8.2 (4.8–13.0)	10.0 (6.7–13.4)	0 (0–13.1)

Abbreviations: JES, Japanese Esophageal Society; TBE, timed barium esophagram.

^a^ Data available.

* *p* < 0.05 on comparison between High‐resolution manometry (HRM)/Chicago Classification achalasia subtypes.

### 3.2 Associations of HRM Subtypes and PEP With Esophageal Width (Brazilian Classification)

49% (*n* = 108) of the cohort had Brazilian class 1 and 46% (*n* = 102) had Brazilian class 2 anatomy on esophagram. Only 5% (*n* = 12) had Brazilian class 3 and none had class 4.

**FIGURE 2:**
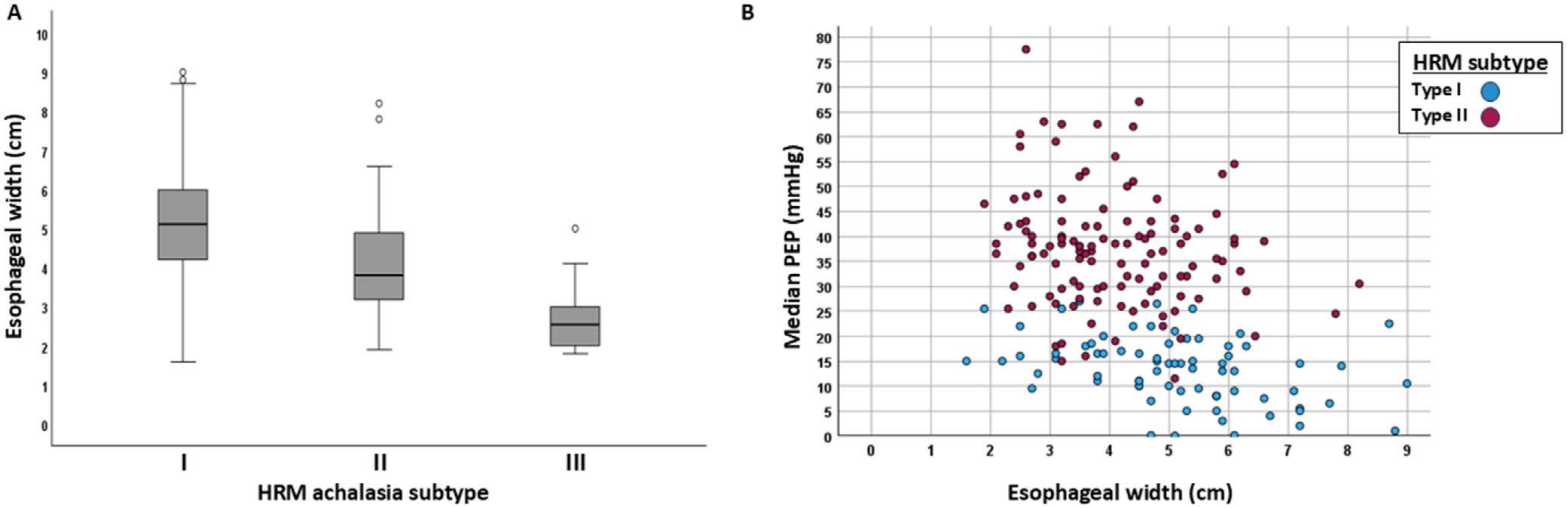
Association of esophageal body width with high‐resolution manometry (HRM) achalasia subtype and pan‐esophageal pressurization (PEP). “Median PEP” is the median of 10 supine swallows on HRM. Esophageal width was measured on esophagram. “^o^” indicates outlier values.

Esophageal width differed between achalasia subtypes (*p* < 0.001), greatest in type I and lowest in type III (pairwise differences between each subtype; adjusted *p*‐values < 0.001); Figure 2. Among patients with type I and type II achalasia, there was also a significant negative correlation between esophageal body width and median PEP (*r* = −0.412; Figure 2) and number of swallows (Figure S2) with PEP > 30 mmHg (*rho =* −0.381), PEP > 20 mmHg (*rho =* −0.392), PEP > 15 mmHg (*rho =* −0.364), and PEP > 10 mmHg (*rho =* −0.396); all *p*‐values < 0.001. There were 16 patients with at least 1 swallow PEP > 70 mmHg; among these, the median (IQR) esophageal width was 3.3 (2.6–4.3) cm.

**FIGURE 3:**
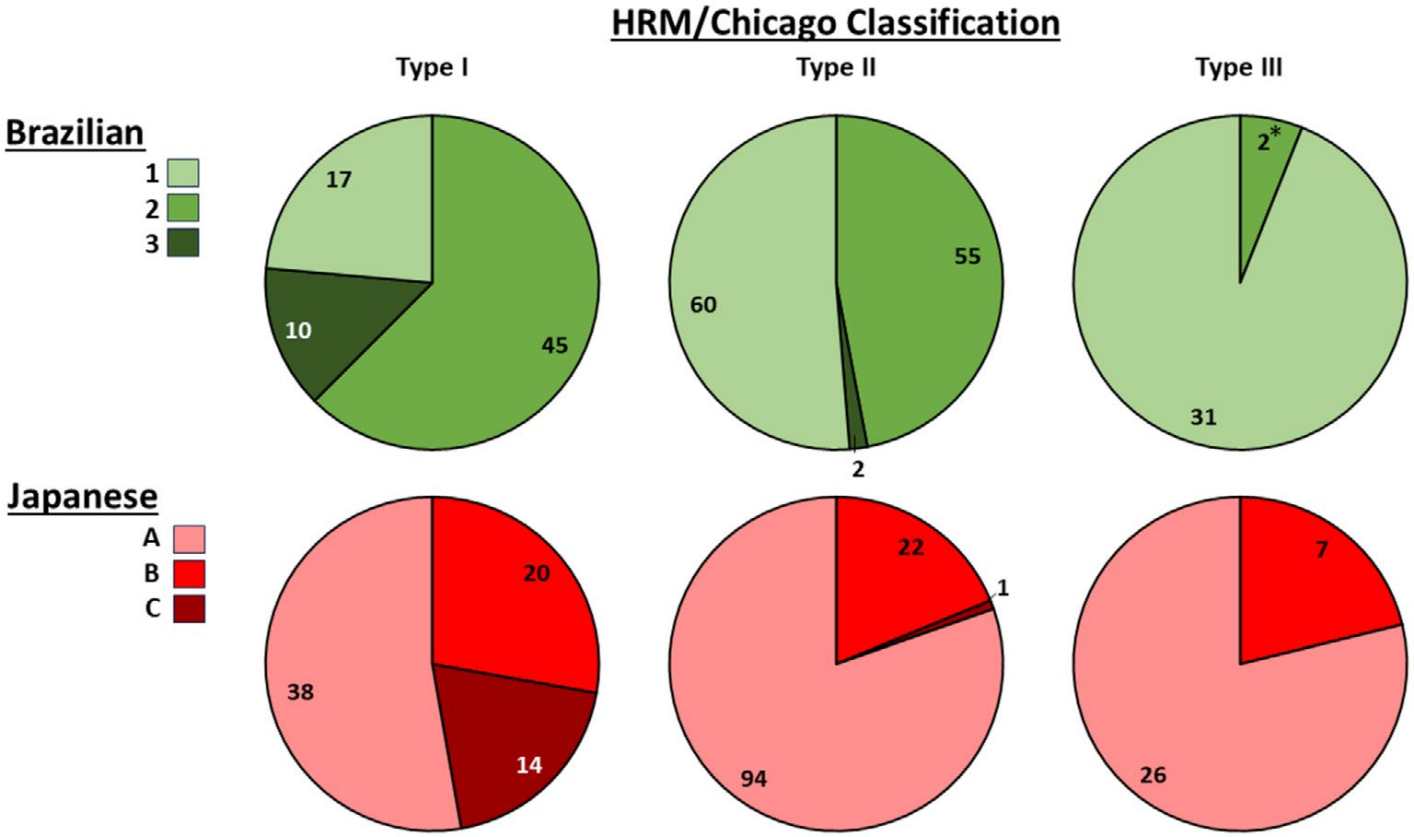
Association of high‐resolution manometry (HRM) achalasia subtypes with esophagram classifications. Data labels represent the number of patients. There were 0 Brazilian class 4 patients in the total cohort. * The two type III achalasia patients with Brazilian class B had esophageal widths of 4.1 and 5.0 cm; 0 type III achalasia patients had Brazilian class 3 or Japanese Esophageal Society (JES) class C.

**FIGURE 4:**
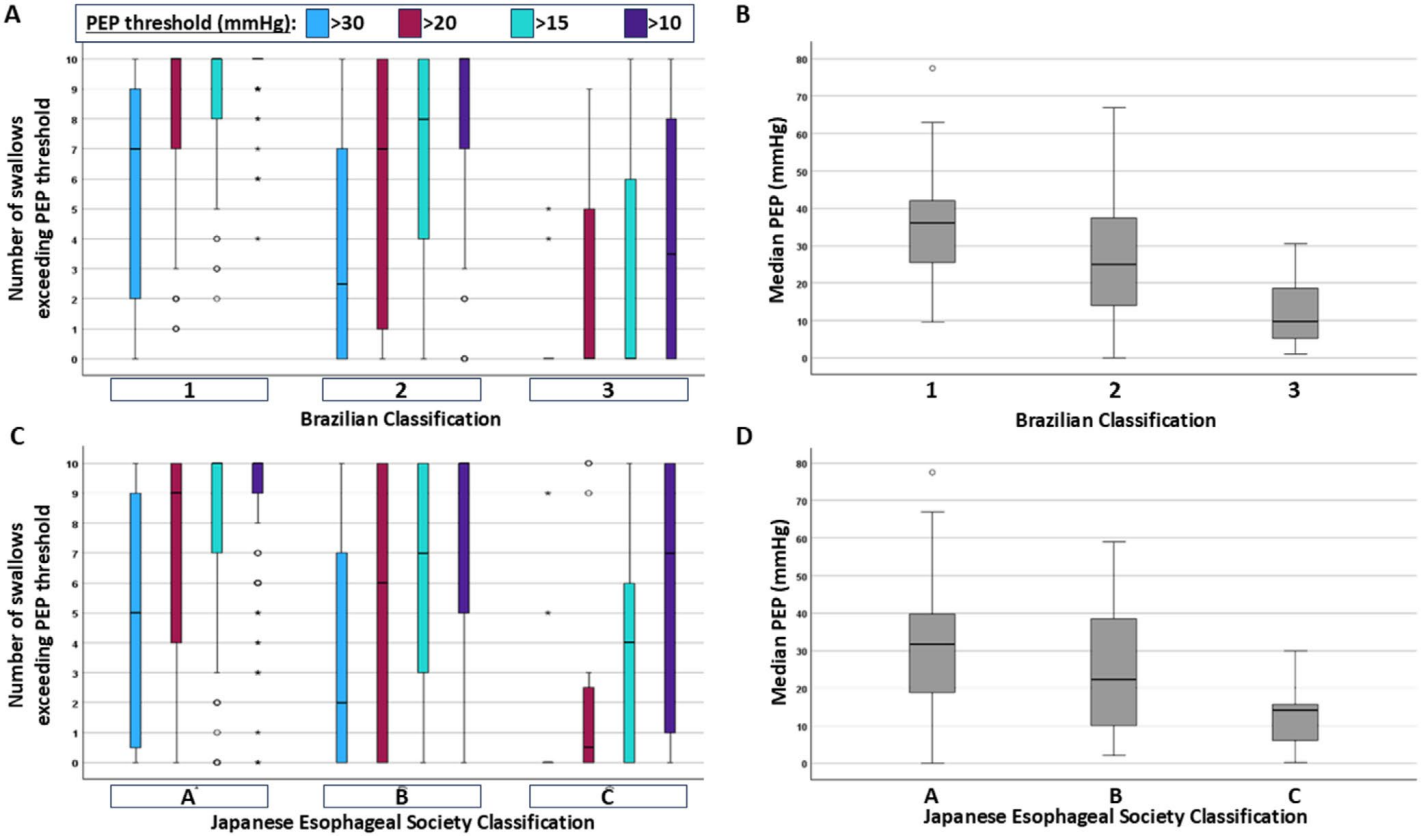
Association with pan‐esophageal pressurization (PEP) on HRM with esophagram‐based achalasia classifications. (A, B) Brazilian classification. (C, D) Japanese Esophageal Society (JES) classification. “*” and “^o^” indicate outlier values.

There were also differences in HRM achalasia subtypes between Brazilian esophagram classifications (*p* < 0.001); Figure 3. Type I was less frequent than type II, which was less frequent than type III achalasia among Brazilian class 1. Among type III achalasia, 94% were Brazilian class 1 and zero were Brazilian class 3. There was otherwise overlap between HRM subtypes and Brazilian classifications. Among patients with type I and type II achalasia on HRM, there also were differences in median PEP and numbers of swallows with PEP > 30, > 20, > 15, and > 10 mmHg between Brazilian classifications (*p*‐values < 0.001; post hoc pairwise adjusted *p*‐values < 0.034); Figure 4.

Among patients with type I achalasia per the Chicago Classification (none had ≥ 2 swallows with PEP > 30 mmHg per criteria), there were 36 (50%) with ≥ 2 swallows with PEP > 20 mmHg, 49 (68%) with ≥ 2 swallows with PEP > 15 mmHg, and 61 (85%) who had ≥ 2 swallows with PEP > 10 mmHg. The frequency of Brazilian classification differed between those with or without ≥ 2 swallows at PEP > 20 mmHg (*p* = 0.007), > 15 mmHg (*p* < 0.001), and > 10 mmHg (*p* = 0.032); Table 2. There were zero patients with Brazilian class A who did not have ≥ 2 swallows with PEP > 15 or 10 mmHg.

**TABLE 2:** Association of pan‐esophageal pressurization (PEP) thresholds among Chicago Classification type I achalasia with Brazilian and Japanese Esophageal Society (JES) classifications.

PEP thresholds	Brazilian			JES		
	1	2	3	A	B	C
≥ 2 swallows > 20 mmHg						
No; *n* (%)	3 (8)	26 (72)	7 (19)	14 (39)	12 (33)	10 (28)
Yes; *n* (%)	14 (39)	19 (53)	3 (8)	24 (67)	8 (22)	4 (11)
≥ 2 swallows > 15 mmHg						
No; *n* (%)	0	16 (70)	7 (30)	7 (30)	9 (39)	7 (30)
Yes; *n* (%)	17 (35)	29 (59)	3 (6)	31 (63)	11 (22)	7 (14)
≥ 2 swallows > 10 mmHg						
No; *n* (%)	0	6 (55)	5 (45)	3 (27)	4 (36)	4 (36)
Yes; *n* (%)	17 (28)	39 (64)	5 (8)	35 (57)	16 (26)	10 (16)

*Note:* By classification criteria, all patients with type I achalasia had < 2 swallows at PEP > 30 mmHg.

Among the 16 patients with at least 1 swallow PEP > 70 mmHg, 11 (69%) were Brazilian class 1 and 5 (31%) were Brazilian class 2.

### 3.3 Associations Between HRM Subtypes and PEP and Esophageal Morphology (JES Classification)

71% (*n* = 158) of the cohort had JES class A, while 22% (*n* = 49) had class B and 7% (*n* = 15) had class C. The association of Brazilian classification and JES classifications is displayed in Table 3.

**TABLE 3:** Association of Brazilian and Japanese Esophageal Society (JES) classifications.

Brazilian classification	JES classification		
	A	B	C
1	94	13	1
2	61	32	9
3	3	4	5

*Note:* The number in each cell represents the number of patients.

There were differences in HRM achalasia subtypes between JES esophagram classifications (*p* < 0.001), Figure 3, though JES class A was the most common JES classification for all three HRM achalasia subtypes. 93% of JES class C patients had type I achalasia on HRM while there were 0 type III achalasia with JES class C.

Among patients with type I and type II achalasia on HRM, there also were differences in median PEP and numbers of swallows with PEP > 30, > 20, > 15, and > 10 mmHg between JES classifications (*p*‐values < 0.001); Figure 4. On post hoc testing, there were pairwise differences between JES A and B, A and C, and B and C (adjusted *p*‐values 0.0–0.049) for each, except for the number of swallows with PEP > 30 mmHg for JES A versus B (adj *p* = 0.072) and the number of swallows > 10 mmHg for JES B versus C (adjusted *p*‐value = 0.203). Among patients with type I achalasia per the Chicago Classification, the frequency of JES classification differed between those with or without ≥ 2 swallows at PEP > 20 mmHg (*p* = 0.050), > 15 mmHg (*p* < 0.032), but a difference was not detected for > 10 mmHg (*p* = 0.145); Table 2.

Among the 16 patients with at least 1 swallow PEP > 70 mmHg, 13 (81%) were JES class A, 3 (19%) were JES class B, and 0 were JES class C.

For other morphologic features on esophagram, there were 12 patients (5% of the total cohort) with a corkscrew appearance on esophagram: 9/12 were type III achalasia on HRM (9/34, 26% of type III achalasia) and 3/12 were type II achalasia (3/119, 2% of type II achalasia; one had swallows (2) with PEP > 70 mmHg); 0 were type I achalasia. There were 24 patients with a sigmoid esophagus: 19/24 were type I achalasia on HRM (19/73, 26% of type I achalasia) and 5/24 were type II achalasia (5/119, 4% of type II achalasia); 0 were type III achalasia.

## 4 Discussion

The major findings of this study were the demonstration of the significant association between esophageal pressurization and achalasia subtype on HRM and the esophageal anatomy assessed on esophagram. Of the HRM subtypes, type I achalasia was the most commonly associated with anatomical distortion, both in terms of dilatation and morphology, that is, greatest width and more advanced Brazilian and JES classifications, while type III achalasia was associated with minimal esophageal dilatation (most Brazilian class 1 and JES‐A). Despite the observed correlations, there was significant heterogeneity of the esophagram classifications across the achalasia subtypes. For example, some patients with low PEP had minimal anatomic change, and vice versa. These findings suggest the potential role for complementary application of HRM and esophagram to provide a more comprehensive functional and structural phenotyping of achalasia.

Prior studies have emphasized the prognostic implications of HRM subtypes, with type II achalasia associated with the highest likelihood of treatment success, and type III achalasia the lowest [1, 12, 13, 14]. The outcome prediction with the specific JES or Brazilian esophagram classifications is less thoroughly described than the Chicago Classification, though sigmoid esophagus was associated with higher rates of treatment failure in previous studies [5, 15]. While the association of esophageal dilatation (based on endoscopic assessment) was assessed in the initial HRM subtype description, and type I achalasia HRM pattern was associated with sigmoid morphology, the relationships between HRM achalasia classifications and degrees of PEP have not previously been assessed in detail as in the current study [1, 5].

While type I achalasia was associated with greater esophageal width and the majority of JES class C, it was also notable that there was variation in esophageal width and anatomic morphology within each HRM subtype, as well as overlap between the achalasia subtypes, particularly between type I and II. While PEP is a finding on HRM supportive of EGJ outflow obstruction, it also relates to interactions between esophageal emptying, esophageal wall mechanics and morphology, and likely the presence or absence of non‐occluding esophageal contractions [1, 6, 16, 17, 18]. Hence, not only were differences (as well as overlap) in esophageal dilatation and morphology on esophagram observed between the HRM subtypes, we also demonstrated differences with detailed examination of the degree of PEP and by number of swallows at various thresholds. That is, among patients with type I and II achalasia on HRM, greater esophageal width (Brazilian class) and anatomical distortion (JES class) were inversely correlated with the magnitude of PEP. This suggests a continuum of achalasia disease severity that spans across our concepts of type I and II achalasia, noting that the thresholds for criteria to differentiate type I from type II (i.e., PEP with a 30 mmHg isobaric contour in ≥ 20% of supine test swallows) were somewhat arbitrarily defined based on pattern recognition [1, 2, 3, 4]. While refining the PEP threshold was not a specific aim of this study, critical evaluation of PEP thresholds (degree of PEP and proportion of swallows) relative to achalasia outcomes is an anticipated future direction, which may better reflect important features of esophageal wall mechanics and remodeling effects that impact outcomes in achalasia.

Type III achalasia, which is signified by the presence of premature (spastic) contractions, was represented anatomically by the lowest esophageal width and infrequent advanced Brazilian or JES classes as there were zero type III achalasia patients with Brazilian class 3 or JES class C. This is intuitive as lumen‐occluding contraction required to generate the contraction‐associated pressure signal on the HRM catheter would unlikely be mechanically feasible in a significantly dilated esophagus. Further, although ‘corkscrew’ morphology has traditionally been associated with spastic esophageal disorders, this was observed in only 26% of the type III achalasia patients in this cohort, while 75% of esophagrams with a corkscrew morphology were type III achalasia on HRM. This suggests corkscrew esophagus as neither an adequately sensitive nor specific finding. Further, high PEP pressures (> 70 mmHg) among those with type II achalasia were previously proposed to potentially reflect a ‘spastic variant’. While only 6% (1/16) of these patients in this study had a corkscrew morphology on esophagram, they otherwise appeared to have minimal esophageal distortion on esophagram with 69% Brazilian class 1 (none class 3 or 4) and 81% being JES class A (none being JES class C).

The study carries strengths related to the comprehensive and detailed analysis of HRM and esophagram from a relatively large cohort of treatment‐naïve achalasia. However, there are also associated limitations. The study cohort is from an academic achalasia referral center, which may limit generalizability and warrants further validation from external cohorts. Further, the study is limited to the pretreatment stage of achalasia, though the cohort was selected based on the availability of post‐treatment outcome data, and an additional study evaluating treatment outcome prediction in this cohort is being pursued.

In conclusion, this study highlights the association between the achalasia subtypes and pressurization on HRM and esophageal anatomy on esophagram in patients with achalasia. Our findings suggest that esophageal structural changes, such as dilatation and tortuosity, correlate inversely with the generation of intraluminal pressure during HRM, which may reflect the chronic remodeling changes associated with the achalasia disease process. However, there was also overlap and variability between classifications that underscore the potential for benefit from a multimodal approach to staging disease severity in achalasia. While additional study is anticipated to further evaluate the impact of these features on treatment outcomes in achalasia, we hypothesize that incorporating HRM‐based features of motility and pressurization with anatomic features provided by radiographic imaging may improve our understanding of achalasia pathophysiology and enhance clinical stratification to guide treatment strategies.

## Author Contributions

J.E.P. contributed to the study concept and design, obtaining funding, data interpretation, editing the manuscript critically, and approval of the final version. E.G., J.M.S., D.A.F., and L.K. contributed to data analysis, data interpretation, and approval of the final version. W.R. contributed to data analysis, critical editing of the manuscript, and approval of the final version. D.A.C. contributed to the study concept and design, obtaining funding, drafting of the manuscript, data analysis, data interpretation, and approval of the final version.

## Conflicts of Interest

D.A.C.: Medtronic (Speaking, Consulting, License); Diversatek (Consulting); Braintree (Consulting); Medpace (Consulting); Phathom Pharmaceuticals (Speaking, Consulting); Regeneron/Sanofi (Speaking). J.E.P.: Sandhill Scientific/Diversatek (Consulting, Grant), Takeda (Speaking), Astra Zeneca (Speaking), Medtronic (Speaking, Consulting, Patent, License), Torax/Ethicon (Speaking, Consulting), EndoGastric Solutions (Advisory Board), Phathom (Speaking, Consulting). Other authors have no conflicts to disclose.

## Supporting information


**Appendix S1:** nmo70180‐sup‐0001‐AppendixS1.docx.

## Data Availability

The data that support the findings of this study are available from the corresponding author upon reasonable request and completion of necessary privacy and ethical approvals.
