# Supplementary material for: Functional‐Structural Correlates in Achalasia: The Relationship of Esophageal Pressurization and Anatomy
Source: Neurogastroenterol Motil. 2025 Oct 9;37(12):e70180. doi: 10.1111/nmo.70180 (PMC12623264; doi:10.1111/nmo.70180)

**Supplemental material**

**Figure S1. Patient flow.** HRM – high-resolution manometry; POEM – PerOral Endoscopic Myotomy; LHM – laparoscopic Heller’s myotomy; TBE – timed barium esophagram

**
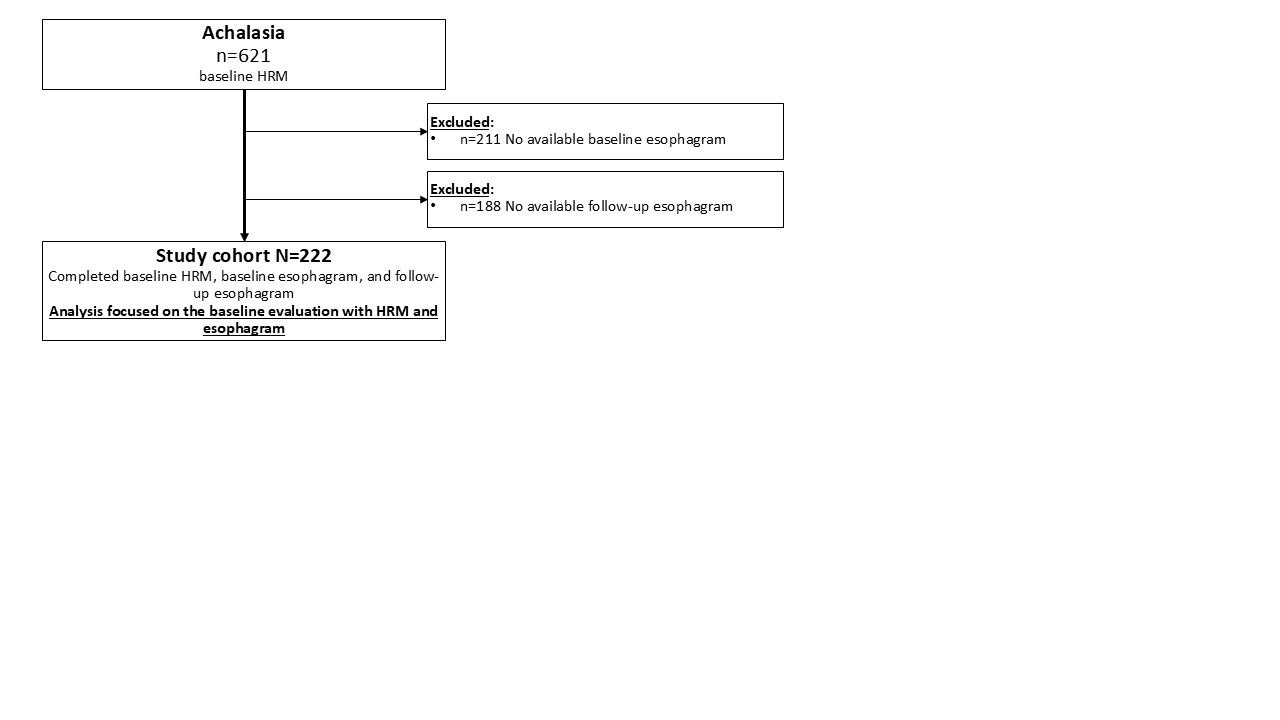
**

**Figure S2. Association of esophageal body width with pan-esophageal pressurization on HRM.** The Y-axis in each plot reflects number of swallows greater than a given PEP threshold: A) >30 mmHg, B) >20 mmHg, C) >15 mmHg, D) >10 mmHg.


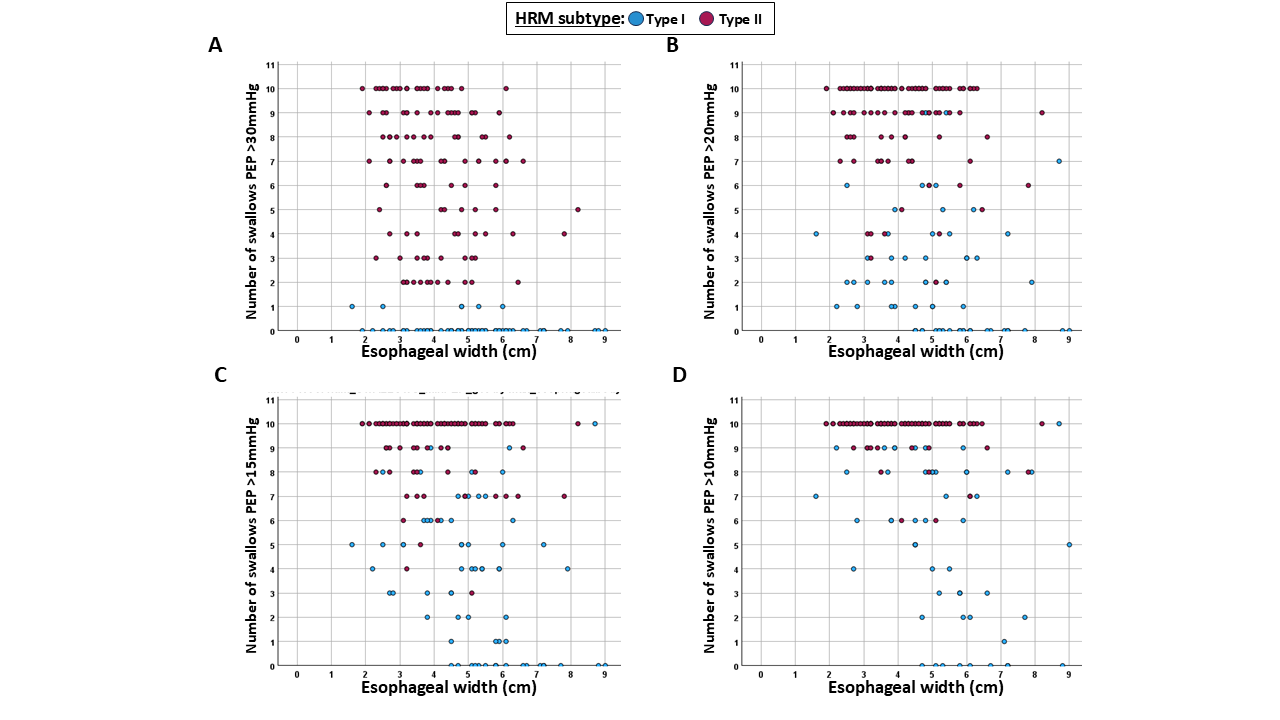

Supplement: Supplementary file 1 — Appendix S1: nmo70180‐sup‐0001‐AppendixS1.docx. [file NMO-37-e70180-s001.docx]
